# Kyste hydatique du foie rompu dans la veine cave inférieure

**Published:** 2011-05-25

**Authors:** Said Ait Laalim, Karim Ibn Majdoub Hassani, Imane Toughai, Abdelmalek Oussaden, Imane Kamaoui, Khalid Mazaz, Khalid Ait Taleb

**Affiliations:** 1 Département de chirurgie générale, CHU Hassan II, Fès, Maroc; 2 Département de Radiologie, CHU Hassan II, Fès, Maroc

**Keywords:** Kyste hydatique hépatique, Rupture, Veine cave inférieure, Chirurgie, Contrôle vasculaire, Maroc

## Abstract

La rupture du kyste hydatique dans la veine cave inférieure est une complication rare et grave des kystes hydatiques hépatique. La manifestation la plus fréquente est l'embolie pulmonaire. L'hémorragie aigue intra-kystique survienne surtout en per-opératoire et elle est d’évolution dramatique. Le diagnostic est basé sur le scanner. Le traitement est chirurgical. Nous rapportons un cas clinique rare chez un patient de 38 ans, opéré en urgence pour un sepsis sur un kyste hydatique du foie comprimant la veine cave inférieur (VCI) avec thrombose partielle de cette dernière. Après évacuation du kyste, une fistulisation spontané per opératoire s'est produite dans la VCI ayant causé le décès du patient. La rupture du kyste hydatique du foie dans la VCI doit toujours être redoutée devant un kyste hydatique des segments postérieurs du foie droit (VII et VIII), comprimant la VCI avec présence en son sein d'une thrombose ou des vésicules filles. Le traitement chirurgical doit être réalisé avec prudence et toujours sous contrôle vasculaire.

## Introduction

L'hydatidose est une maladie parasitaire endémique due à l’*Echinococcus granulosus* [1]. Le chien est l'hôte définitif et l'homme l'hôte intermédiaire accidentel dans le cycle de vie de ce tænia [1]. La rupture dans la veine cave inférieure (VCI) est une complication rare et grave du kyste hydatique du foie (KHF). Nous rapportons l'observation d'une fistulisation spontanée per opératoire d'un kyste hydatique hépatique dans la veine cave inférieure

## Observation

Patient de 38 ans, originaire du Nord Est marocain, est admis au service des urgences pour douleurs fébrile de l'hypochondre droit. Il a été opéré il y a 5 mois dans un hôpital périphérique pour KHF droit sans pouvoir réséqué le kyste.

L'examen général trouve un patient fébrile à 39 °c, une tachycardie à 92 bat/mn, une tachypnée à 24C/mn avec un état hémodynamique stable. L'examen abdominal trouve une légère sensibilité de l'hypochondre droit. L'auscultation pleuro-pulmonaire note une diminution des murmures vésiculaires de la base pulmonaire droite. L'examen cardio-vasculaire est sans particularité. Biologiquement on note une hyperleucocytose à 22000/mm^3^, une insuffisance rénale fonctionnelle avec une urée à 0,85g/l, une créatinine normale et une élévation de la CRP à 217mg/l.

**Figure 1:**
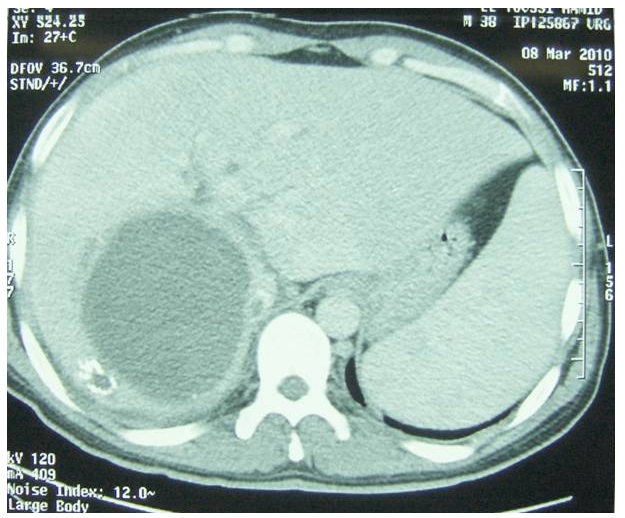
Tomodensitométrie Abdominale, coupe axiale: kyste hydatique du foie type I de siège postérieur droit. La veine cave inférieure est comprimée et partiellement thrombosée

**Figure 2:**
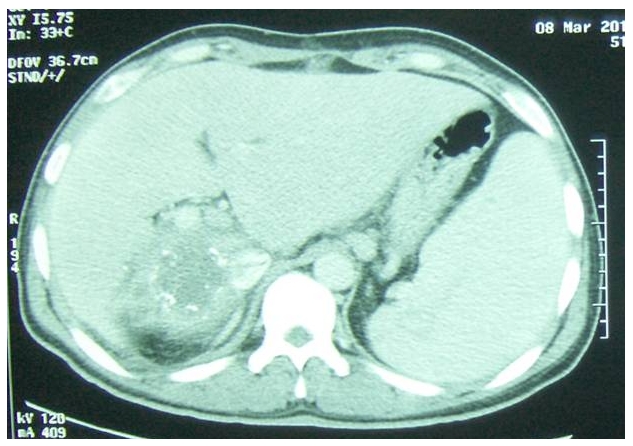
Tomodensitométrie Abdominale, coupe axiale: KHF calcifié avec des calcifications périphériques et thrombose partielle de la veine cave inférieure

La radiographie du thorax est sans particularité et au scanner abdominal on trouve la présence d'un volumineux kyste hydatique hépatique de 10/08 cm de diamètre intéressant les segments I, VII et VIII type I, la VCI est comprimée par la formation kystique et partiellement thrombosée. (Figure 1). Il existe une hypertrophie du foie gauche ainsi que du segment V hépatique avec individualisation au sein du kyste de quelques calcifications linéaires (Figure 2).

Le diagnostic retenu est celui d'un sepsis sévère sur KHF infecté, associé à une thrombose partielle de la VCI. La conduite à tenir est de mettre le patient sous anticoagulant à dose curative, de drainé chirurgicalement le kyste en urgence et instauré ultérieurement un traitement médical à base d'Albendazole.

L'exploration per-opératoire trouve la présence d'un KH des segments I, VII et VIII du foie symphysé au diaphragme, sans possibilité de libération du kyste. L'ouverture du kyste est réalisée à travers une partie saillante et libre du kyste avec aspiration d'un liquide trouble sans vésicules filles (KH type I), dans le même temps opératoire on a constaté une extériorisation spontanée de la membrane proligère, rapidement suivi d'un saignement aigue et foudroyant sans pouvoir localisé l'origine du saignement ni de faire l'hémostase. Le patient a rapidement instauré une asystolie irrécupérable. L'examen anatomique révèle une ouverture quasi complète du kyste hydatique sur la face antérieur de la VCI.

## Discussion

Le kyste hydatique du foie est une affection parasitaire due au développement de la forme larvaire du tænia du chien *Echinococcus granulosus*. Cette pathologie demeure fréquente et constitue un problème de santé publique dans les pays de forte endémie. Les aspects cliniques du KHF sont très divers. Les complications infectieuses, biliaires et thoraciques sont présentes dans 40% des cas [2]. La rupture spontanée du KHF dans la VCI est très rare seulement une douzaine de cas ont été rapportés dans la littérature. La principale symptomatologie est l'embolie pulmonaire, elle est rarement décrite et le plus souvent découverte à l'autopsie [3, 4]. Les données per-opératoire et post-mortem indiquent que l'embol est du à une obstruction purement mécanique par le kyste et les vésicules filles. Il n'existe aucun stigmate de caillot ou de thrombose associés [4]. Théoriquement le choc hémorragique par rupture spontanée du KHF dans la VCI devrait être la principale complication, cependant ça n'a jamais été décrit dans la littérature et ceci peut être expliquée probablement à ce que ces malades décèdes avant leur arrivés à l'hôpital. Chez notre patient, le diagnostic retenu a été celui d'un sepsis sévère sur un KHF infecté, sans toutefois pouvoir éliminer une embolie pulmonaire.

Sur le plan radiologique le scanner reste le meilleur examen qui permet de préciser les rapports vasculaire du KHF notamment avec la VCI [5]. Sur cet examen la rupture du KHF dans la VCI est suspectée devant la présence d'un kyste hydatique des segments postérieur du foie (VII, VIII et I) et au contact de la VCI. Cette dernière peut être comprimée, laminée par le kyste avec présence parfois en son sein d'une thrombose ou de matériels hydatique. L'IRM, l’échographie Doppler et la cavography permettent de confirmer la présence de lésions kystiques dans la veine cave rétro-hépatique et dans l′artère pulmonaire [6]. Chez notre patient le scanner avait permis de montré la présence d'un KH des segments postérieurs du foie droit, comprimant la VCI avec thrombose partielle de cette dernière.

Le traitement du KHF rompu dans la VCI est chirurgical. Deux principales complications sont à redoutées en per opératoire, l'embolie pulmonaire per opératoire massive et l'hémorragie foudroyante, c'est la raison pour laquelle un contrôle vasculaire préalable est indispensable avant tout acte chirurgical [6, 7]. Le kyste hydatique doit être manipulé avec précaution, il faut chercher la communication kysto-veineuse et la fermé. L'absence de sang dans la cavité kystique n’élimine pas une rupture du KHF dans la VCI. Ceci a été expliqué par le faite que la pression intra-kystique est supérieure à la pression intraveineuse [6]. Notre cas viens appuyer cette théorie vue qu'on per opératoire et à l'ouverture du kyste il n'y avait aucune stigmate de saignement intra-kystique.La cure de l'embolie pulmonaire hydatique peut être réalisée dans le même acte opératoire ou à distance. L'extraction des vésicules filles du tronc de l'AP et de ses branches proximales se fait sous circulation extra corporelle (CEC) par artériotomie [5]. Le traitement médical est indiqué dans les embolies chroniques, l'essaimage important de vésicules filles intéressant tout l'arbre artériel pulmonaire ou en cas de contre-indication de la chirurgie [5].

## Conclusion

La rupture du KHF rompu dans la VCI est une complication rare et grave. Le scanner est l'examen de choix qui permet le diagnostic de cette complication. L'embolie hydatique pulmonaire reste la complication la plus fréquente. Le traitement du KHF rompu dans la VCI est chirurgical et doit être réalisée sous contrôle vasculaire. Le pronostic est réservé.
